# Hydrochemistry and Dissolved Inorganic Carbon (DIC) Cycling in a Tropical Agricultural River, Mun River Basin, Northeast Thailand

**DOI:** 10.3390/ijerph16183410

**Published:** 2019-09-14

**Authors:** Xiaoqiang Li, Guilin Han, Man Liu, Chao Song, Qian Zhang, Kunhua Yang, Jinke Liu

**Affiliations:** 1 Institute of Earth Sciences, China University of Geoscience (Beijing), Beijing 100083, China; 2 The Institute of Hydrogeology and Environmental Geology, Chinese Academy of Geological Sciences, Shijiazhuang 050061, China; 3 Institute of Geographic Sciences and Natural Resources Research, Chinese Academy of Sciences, Beijing 100101, China

**Keywords:** stable carbon isotope, major elements, dissolved inorganic carbon, agriculture, Mun River Basin, Northeast Thailand

## Abstract

Dissolved inorganic carbon isotope composition (δ^13^C_DIC_), together with major ion concentrations were measured in the Mun River and its tributaries in March 2018 to constrain the origins and cycling of dissolved inorganic carbon. In the surface water samples, the DIC content ranged from 185 to 5897 μmol/L (average of 1376 μmol/L), and the δ^13^C_DIC_ of surface water ranged from −19.6‰ to −2.7‰. In spite of the high variability in DIC concentrations and partial pressure of carbon dioxide (*p*CO_2_), the δ^13^C_DIC_ values of the groundwater were relatively consistent, with a mean value of −16.9 ± 1.4‰ (*n* = 9). Spatial changes occurred in the direction and magnitude of CO_2_ flux through water-air interface (*F*_CO2_). In the dry season, fluxes varied from −6 to 1826 mmol/(m^2^·d) with an average of 240 mmol/(m^2^·d). In addition to the dominant control on hydrochemistry and dissolved inorganic carbon isotope composition by the rock weathering, the impacts from anthropogenic activities were also observed in the Mun River, especially higher DIC concentration of waste water from urban activities. These human disturbances may affect the accurate estimate contributions of carbon dioxide from tropical rivers to the atmospheric carbon budgets.

## 1. Introduction

The global estimate of atmospheric carbon dioxide exchange shows that the amount of carbon dioxide exchanged in the tropics is huge and cannot be ignored [1]. At present, because of the role of atmospheric carbon dioxide in controlling global climate change, the process of controlling carbon in and out of inland water is receiving special attention [2,3,4,5]. As an important part of inland water, river, and groundwater are the key to the hydrogeology of the upper crust and the surface of the earth [6,7,8,9,10]. Many scholars have begun to undertake systematic research on rivers, especially the conversion transfer of CO_2_ among the lithosphere, hydrosphere, and the atmosphere systems [5,11,12,13,14,15]. However, the special connection between hydrological and biogeochemical processes regulating carbon dioxide fluxes in above systems is still unclear. Some studies have shown that dissolved inorganic carbon (DIC) accounts for about 50% of global carbon fluxes transported by the rivers [16]. In addition, a huge amount of DIC is not only transported by the river into the ocean, but also released into the atmosphere [17,18,19,20]. An updated estimate of global CO_2_ evasion to the atmosphere from inland waters is about 2.1 Pg C/year [1]. Thus, both vertical and lateral transports of DIC via rivers should be further understood.

The DIC in river water mainly originates from the following processes of CO_2_ exchange with the atmosphere, inflow of soil CO_2_ through groundwater, biological respiration in the river, and dissolution of carbonate rocks [21]. Moreover, human factors also affect all aspects of the carbon cycle in a river. For instance, flow regulation and damming affect the basic hydraulic characteristics of the river (water flow velocity and mixing characteristics), and cut off the river’s connection with adjacent areas. Frequent exchange between groundwater and river water increases the risk of water pollution, because possible contaminants from human activities could be carried into the groundwater. High contents of organic carbon in waste water can lead to mass microbial growth, which make groundwater is unsuitable for residents. Understanding the DIC cycle in rivers and streams helps in predicting the behavior of the river system in case of contamination, and in assessing the possible contaminating effect of the river water on groundwater.

The Mun River, in northeast Thailand, has a considerable amount of population and agriculture, therefore, the risk of such contamination is also elevated. Groundwater in the area is used as drinking water and irrigation water. For all these reasons, the Mun River is an ideal example of a vulnerable river system characterized by significant human activity. Under the right conditions, the carbon isotope of DIC (δ^13^C_DIC_) can be an effective tool, and it is helpful to understand the biogeochemical reactions processes in surface water and groundwater. Correlation of variations in δ^13^C_DIC_ with major ion chemistry, the partial pressure of carbon dioxide (*p*CO_2_), and calcite saturation indexes (CSI) may provide evidence for such processes.

The objective of this study was to discriminate various DIC sources, especially anthropogenic sources in a tropical agricultural river using isotopic and chemical tracers. For this, this paper combines δ^13^C values of DIC and hydrochemistry to constrain the origins and cycling of DIC in the Mun River of Northeast Thailand. This study characterizes DIC sources and interactions in a tropical agricultural river system, and thus helping to increase the overall understanding of the global carbon cycle and the links among the lithosphere, hydrosphere, and the atmosphere.

## 2. Materials and Methods

### 2.1. Study Area

**Figure 1:**
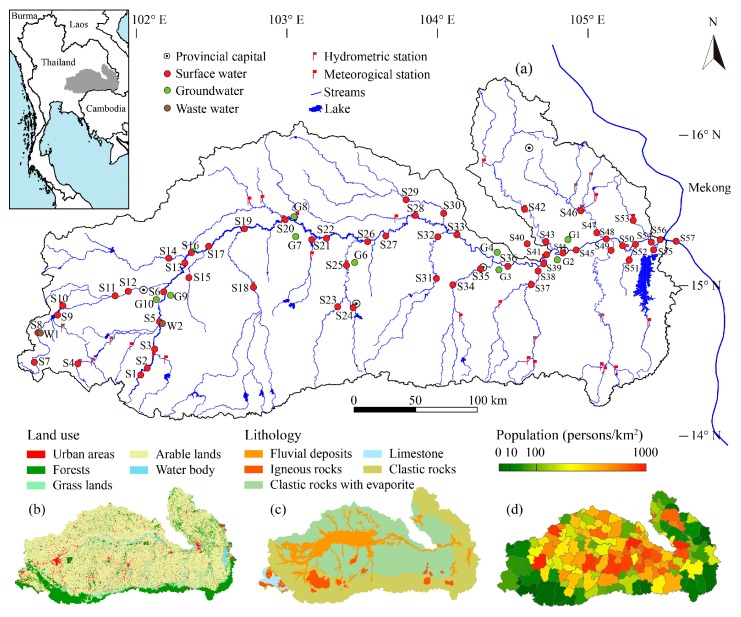
Maps of the Mun River Basin created by using ArcGIS software. (**a**) The hydrographic network with sampling sites and (**b**) land uses in the Mun river basin; (**c**) lithology of the Mun river basin; (**d**) density of population at district level (persons per square kilometer).

The study area and sample sites were described in detail by the authors of references [22,23]. Briefly, the Mun River lies in the northeast of Thailand between latitudes 14° N to 16° N and longitudes 101°30′ E to 105°30′ E (Figure 1a), the total area is 71,060 square kilometers. It is one of the right tributaries of the Mekong River [24,25]. Thailand is a traditional agricultural country, and agriculture is the largest sector of the economy. Arable land (70.8%) is the main land use type in the study area and others such as forest land (13.5%), grassland (5.3%), urban (including industrial and residential) areas (6.4%), and water body (4.0%) make up a very small percentage (Figure 1b). Most of the arable land (about 75%) in the Mun River Basin is devoted to paddy fields [26]. The study area is mainly occupied by clastic sedimentary rocks of Mesozoic and Quaternary sediments (Figure 1c). Quaternary sediments are mainly semi-consolidated and unconsolidated sediments. There is a small amount of volcanic rock in the south of the basin. Nevertheless, the sum of all limestones is less than 1% of the bedrock geology of the Mun River basin. Thailand has a population of 67 million, of which one third lives in the Northeast, but this area contributes only 10% to the national GDP [27]. The river or stream flows through several urban centers, where the wastewater is not adequately treated. According to government statistics, the population in the study area is mainly concentrated in the middle reaches, and the distribution range is 37 to 601 persons/km^2^ with an average of 150 persons/km^2^ in the whole basin (Figure 1d).

**Figure 2:**
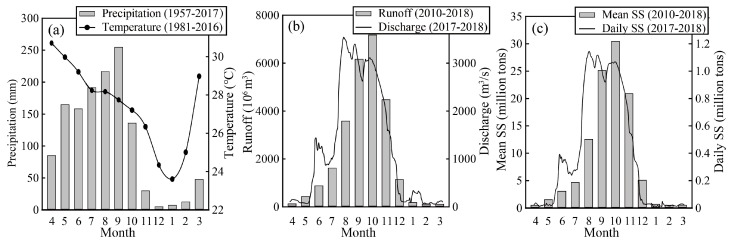
(**a**) Monthly distribution of precipitation (1957 to 2017) and air temperature (1981 to 2016). (**b**) Multiyear (2010–2018) average runoff and daily discharge in gauging station (M.11B) at the outlet of the basin. (**c**) Multiyear (2010–2018) average suspended sediment (SS) and daily suspended sediment in gauging station (M.11B) at the outlet of the basin.

The climate of the basin is under the influence of southwest monsoon from mid-May to mid-October and northeast monsoon from mid-October to mid-February. From mid-February to mid-May, this is the transitional period from the southwest to northeast monsoons. The average annual precipitation is 1308 mm with an increasing trend from upstream (1035 mm) to downstream (1616 mm), and the precipitation in the southwest monsoon season (rainy season) accounts for 85.2% of the annual precipitation (Figure 2a). The average monthly temperature throughout the basin ranges from 23.6 °C in January to 30.7 °C in April. The average annual runoff and annual suspended sediment (SS) at the M.11B hydrological station located at outlet of the Mun River are 2.6 × 10^10^ m^3^ and 1.1 × 10^8^ t (Figure 2a,b) (Royal Irrigation Department Thailand, http://hydro-4.rid.go.th).

### 2.2. Water Sampling and Analysis

All samples in this study were collected in March of 2018. Fifty-six surface water and 2 wastewater samples from the Mun river basin were collected in previous washed containers. One of the wastewater samples (W2) was collected from a sewage pipe in the Khorat Province. Another waste sample was (W1) taken from a rice field where compound fertilizer was added. For comparison, 2 river water samples (S40 and S57) from 2 nearby rivers (Chi and Mekong River) were collected for analysis. In addition, 10 groundwater samples were collected and analyzed in March. The groundwaters were sampled from an existing pumping water well, and depths of this wells ranged from 5 to 30 m. At the sampling points, temperature (T), electrical conductivity (EC), pH, total dissolved solids (TDS), dissolved oxygen (DO), and oxidation reduction potential (ORP) of the water samples were measured using a handheld multi-parameter water meter (YSI Inc., Yellow Springs, OH, USA). Alkalinity was determined on site using a pure HCl titration before filtration. Collected water samples filtered through the cellulose acetate member (Millipore, 0.22 μm), then all samples were stored in pre-cleaned HDPE (high-density polyethylene) bottles.

Major cations (${\mathrm{Mg}}^{2+}$, ${\mathrm{Mg}}^{2+}$, $K^{+}$ and ${\mathrm{Na}}^{+}$) were analyzed by ICP-OES (Optima 5300DV, PerkinElmer Inc., Waltham, MA, USA), and anions (${\mathrm{SO}}_{4}^{2-}$, ${\mathrm{Cl}}^{-}$ and ${\mathrm{NO}}_{3}^{-}$) were analyzed using ionic chromatography (Dionex 1100, Sunnyvale, CA, USA) in the Institute of Geographic Sciences and Nature Resources Research, Chinese Academy of Sciences (CAS). Total dissolved inorganic carbon (DIC) in the river included CO_2_(aq), H_2_CO_3_, ${\mathrm{HCO}}_{3}^{-}$, and ${\mathrm{CO}}_{3}^{2-}$ [17]. Carbon has 2 stables isotopes (^12^C and^13^C), and ratios of these isotopes were reported in 10 percentiles relative to the standard Vienna Pee Dee Belemnite (VPDB). The measurements of ^13^C/^12^C_DIC_ were conducted using a Thermo Fisher Scientific Isotope Gas Ratio Mass Spectrometer (MAT 252) located in the state key laboratory of environmental geochemistry in Guiyang. Following standard methods [28,29], about a 10 mL sample was injected into glass bottles with 1mL 85% H_3_PO_4_. Then generated CO_2_ was extracted into a vacuum line in the laboratory at 50 °C, while stirring for 10 min. Finally, the CO_2_ was transferred cryogenically into a tube for isotope measurement, where δ^13^C (‰) = [(^13^C/^12^C) sample/(^13^C/^12^C) VPDB − 1] × 1000. Routine δ^13^C_DIC_ measurements have an overall precision of ± 0.1‰.

In this study, the DIC content, calcite saturation indexes (CSI) and the partial pressure of carbon dioxide (*p*CO_2_) were calculated from the major ion concentrations, water temperature, alkalinity, and pH using the program PHREEQC version 2.2. In the program, CSI was calculated using the equation: CSI = Log (IAP/K). In the equation, K is the equilibrium constant of the calcite dissolution reaction and IAP is the ion activity product. If CSI >0, the mineral was supersaturated to the aqueous solution and may deposit calcite or dolomite; if CSI = 0, the mineral and the aqueous solution were in equilibrium; and if CSI <0, the mineral was not saturated to the aqueous solution.

### 2.3. Data Processing

The map of land use (Figure 1b) was derived from the Ministry of Natural Resource and Environment of Thailand. The geology map (Figure 1c) was based on the “Geological Map of Thailand” (1:250,000); hydrological and geochemical parameters were analyzed by Microsoft Excel (Microsoft, Redmond, WA, USA) and Sigma Plot 12.5 software. In this paper, figures were drawn using Adobe Illustrator CC 2015.3.

## 3. Results

### 3.1. Geochemistry of the Mun River Water

The physical–chemical parameters of the river water and carbon isotope composition of the dissolved inorganic carbon (δ^13^C_DIC_) are listed in the Supplementary material. Surface river water (Mun, Chi, and Mekong River) displayed a temperature range from 24.0 to 33.0 °C (average, 28.6 °C) with pH ranging from 6.1 to 8.5 (average, 7.4). Groundwaters were cooler (24.0 to 24.9 °C), and have a lower pH (4.7 to 7.3, average 6.5) compared with the surface water. Measured TDS in the field showed significant spatial variations from 15 to 1502 mg/L (average, 297 mg/L). From upstream to downstream, TDS increased first and then decreased due to the import of the Chi River and peak appeared in the middle reaches. In the main channel, EC had similar spatial variation characteristics with TDS and from 23 μS/cm to 2452 μS/cm. Compared with TDS and EC, ORP and DO showed small spatial changes. The observed ORP values in most of samples (average of 165 mV) were positive except from one urban waste sample (W2) in the upstream. Urban sewage contained the lowest DO value (1.6 mg/L) of the whole sample. Groundwaters had higher TDS values (36 to 1359 mg/L, average of 598 mg/L) and EC value (47 to 3166 μS/cm, average of 1093 μS/cm). However, ORP and DO in the groundwaters were similar to the surface waters.

**Figure 3:**
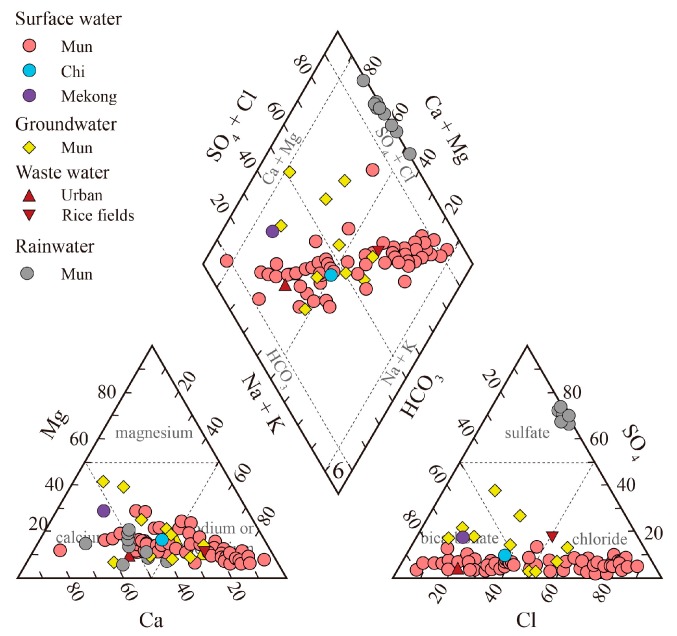
Piper diagram showing cations and anions compositions. The data of the rainwater came from Data report on the acid deposition in the East Asian Region. 2016. Available online: http://www.eanet.asia.

The major ions of surface water and groundwaters were plotted in Figure 3. The average content (μmol/L) of the major ions was in the order of ${\mathrm{Na}}^{+}$ > ${\mathrm{Ca}}^{2+}$ > ${\mathrm{Mg}}^{2+}$ > $K^{+}$ for the cations and ${\mathrm{Cl}}^{-}$ > ${\mathrm{HCO}}_{3}^{-}$ > ${\mathrm{SO}}_{4}^{2-}$ > ${\mathrm{NO}}_{3}^{-}$ for the anions in the surface water samples. The plots indicate that most of the surface and groundwater samples were dominated by ${\mathrm{Na}}^{+}$ for cations and ${\mathrm{Cl}}^{-}$ or ${\mathrm{HCO}}_{3}^{-}$ for anions. The concentration of other cations such as ${\mathrm{Mg}}^{2+}$ and ${\mathrm{Ca}}^{2+}$ was less than 20% of the sum of the major cations. For anions, the sum of ${\mathrm{SO}}_{4}^{2-}$ and ${\mathrm{NO}}_{3}^{-}$ only contributes about 15% of the total anions in the surface and groundwater samples.

### 3.2. DIC System and δ^13^C_DIC_ Values

In the surface water samples, the DIC content ranged from 185 to 5897 μmol/L (average as 1376 μmol/L). In most of the samples, bicarbonate was the main component of DIC, accounting for about 82% of DIC, and the rest was mainly composed of dissolved carbon dioxide as they were more acidic samples. Urban domestic sewage contained abnormally high DIC (7397 μmol/L), and ${\mathrm{HCO}}_{3}^{-}$ was the dominant species (92.4%). Compared with the average of surface water in the same period, farmland water showed lower DIC concentration (632 μmol/L). Chi and Mekong had similar DIC concentrations 1297 μmol/L and 1669 μmol/L, respectively. In groundwaters, the concentrations of DIC (1669 to 17,551 μmol/L, average 8793 μmol/L) were significantly higher than surface water. In most samples, the partial pressure of CO_2_ (*p*CO_2_) was above the atmospheric level (400 ppm). The CSI results of the most of samples were below zero, indicating that the water was undersaturated with CaCO_3_.

The carbon isotopic compositions (δ^13^C_DIC_) of the surface water ranged from −19.6‰ to −2.7‰ in the dry season. Chi and Mekong had similar δ^13^C_DIC_ values to Mun River. Urban sewage and farmland water from rice field did not show abnormal carbon isotope values (−11.4‰ and −13.8‰, respectively). Although the DIC concentration and carbon dioxide partial pressure *p*CO_2_ varied greatly, the δ^13^C_DIC_ values of groundwater samples were relatively consistent, and the average value was −16.9 ± 1.4‰ (*n* = 9) except from G1 (δ^13^C_DIC_ = −9.3‰).

## 4. Discussion

### 4.1. Sources of Groundwater DIC

In the Mun River, *p*CO_2_ values in groundwater were generally higher, more than 10,000 μatm. Groundwater recharge may be the main source of DIC in the surface water, especially in the dry season. Carbonate dissolution mainly occurs in groundwater by reaction with H_2_CO_3_ produced from CO_2_ in the soil rather than the atmosphere [30]. Soil CO_2_ is mainly derived from the respiration of microbial and plant, a minor amount of atmospheric CO_2_ (δ^13^C = −8‰) may be mixed in the shallow layer of the soil [30]. Respiration is a chemical process in which an organism oxidizes and decomposes organic matter in cells and produces energy, at the same time producing carbon dioxide (respired CO_2_) with approximately the same δ^13^C value to soil organic matter [31]. In the study area, the soil organic carbon (SOC) had δ^13^C values of −28‰ to −25‰ (unpublished data), this SOC isotopic range reflects C3 photosynthesis, probably by rice. Most studies have found that due to the different molecular diffusion rates of ^13^CO_2_ and ^12^CO_2_, δ^13^C values of soil CO_2_ is higher than of the organic matter, which is about 4‰ enrichment [32,33,34], the δ^13^C values of carbon dioxide from the oxidation of soil organic matter with δ^13^C (−28‰ to −25‰) will be −24‰ to −21‰ (average, −22‰). Zhao, et al. [35] have reported that the average soil pH value is 6 in the Mun River Basin, thus the predominant carbon species in soil water is carbonic acid [16].

**Figure 4:**
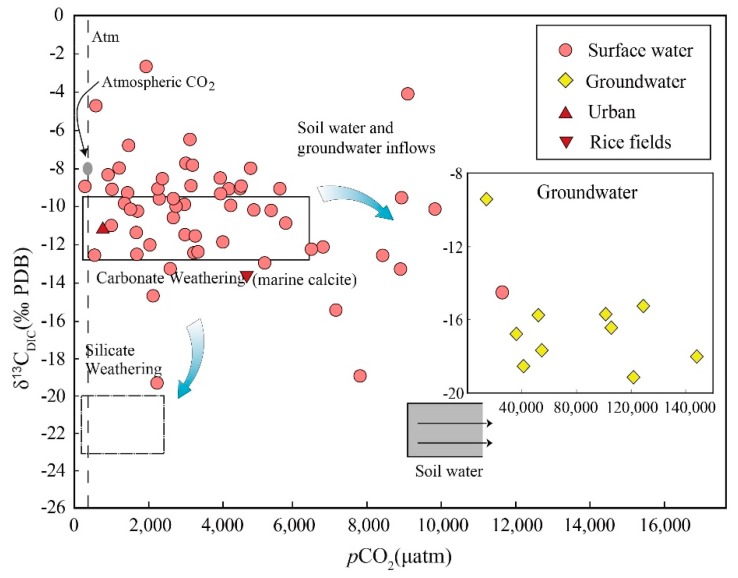
Variation between δ^13^C_DIC_ values and *p*CO_2_. Figure based on (elmer and Veizer [17], Cartwright [20]).

Moreover, the dissolution of soil CO_2_ into soil water produces a further fractionation about 1‰ at 25 °C [17]. Thus, the predicted δ^13^C_DIC_ of the soil water were −23‰ to −20‰ at pH = 6 and 25 °C. Based on the above discussion, it can be inferred that the δ^13^C values of ${\mathrm{HCO}}_{3}^{-}$ from silicate weathering with soil CO_2_ would range from −23‰ to −20‰, which was lower than the measured average value (−16.9 ± 1.4‰) of groundwater. Because δ^13^C of carbonate is almost equal to zero, thus weathering of such carbonates with CO_2_ from soil would produce a positive δ^13^C_DIC_ of −11‰ compared with soil CO_2_. The expected δ^13^C_DIC_ in the process of carbonate weathering was higher than the measured amount. Most of the groundwater samples had δ^13^C_DIC_ values in the range −19.0‰ to −15.1‰, it shows that the DIC in these groundwaters was mainly derived both from the weathering of silicates and carbonates (Figure 4). In addition, the concentration of carbon dioxide in the soil zone was much higher than the atmosphere and groundwater. Under open system, the δ^13^C_DIC_ values of groundwater will tend to more negative due to continued isotopic exchange with carbon dioxide in the soil.

### 4.2. Rock Weathering Versus the δ^13^C_DIC_ Values

Chemical weathering of silicates and carbonates is a key process for the carbon cycling at the Earth’s surface [36,37,38,39,40]. Chemical weathering is the major source of elements delivered by rivers to the oceans [41]. Carbonic acid usually accelerates silicate weathering, for instance, the incongruent dissolution of sodium feldspar to clay kaolinite by carbonic acid:

(1) $$2{\mathrm{NaAlSi}}_{3}O_{8}(s)+9H_{2}O+2H_{2}{\mathrm{CO}}_{3}(\mathrm{aq})={\mathrm{Al}}_{2}{\mathrm{Si}}_{2}O_{5}(\mathrm{OH}{)}_{4}(s)+2{\mathrm{Na}}^{+}\left(\mathrm{aq}\right)+2{\mathrm{HCO}}_{3}^{-}+4H_{4}{\mathrm{SiO}}_{4}\left(\mathrm{aq}\right)$$

As discussed previously, the carbonic acid is most likely to be produced in the soil, where *p*CO_2_ values are much higher than the atmosphere [6,17,20]. Thus, the δ^13^C_DIC_ values in reaction (1) is similar to the estimated value of carbonic acid (−23‰ to −20‰). However, carbonate weathering is much faster than silicate weathering under the same conditions. Consequently, even in many carbonate-poor catchments and minor carbonate mineral weathering may also occur [42,43,44]:

(2) $${\mathrm{CaCO}}_{3}+H_{2}O+{\mathrm{CO}}_{2}(\mathrm{aq})={\mathrm{Ca}}^{2+}\left(\mathrm{aq}\right)+2{\mathrm{HCO}}_{3}^{-}\left(\mathrm{aq}\right)$$

There is only a small amount of carbonate in the upper reaches of the Mun River, these marine carbonates probably have δ^13^C_DIC_ values close to 0‰. Weathering of such carbonate via reaction (2) would produce DIC with a δ^13^C_DIC_ value of −12‰ to −10‰. These predicted δ^13^C_DIC_ values are matched to the value of δ^13^C_DIC_ in the dry season. Although H_2_CO_3_ weathering is common, it is not the only one. Anthropogenic acid (sulfuric and nitric acid) also actively participates in the rock weathering process [7,45,46,47]. For example, anthropogenic emissions of SO_2_ from coal combustion will produces H_2_SO_4_ that can then accelerate carbonate weathering:

(3) $${\mathrm{CaCO}}_{3}+H_{2}{\mathrm{SO}}_{4}=2{\mathrm{Ca}}^{2+}\left(\mathrm{aq}\right)+2{\mathrm{HCO}}_{3}^{-}\left(\mathrm{aq}\right)+{\mathrm{SO}}_{4}^{2-}$$

**Figure 5:**
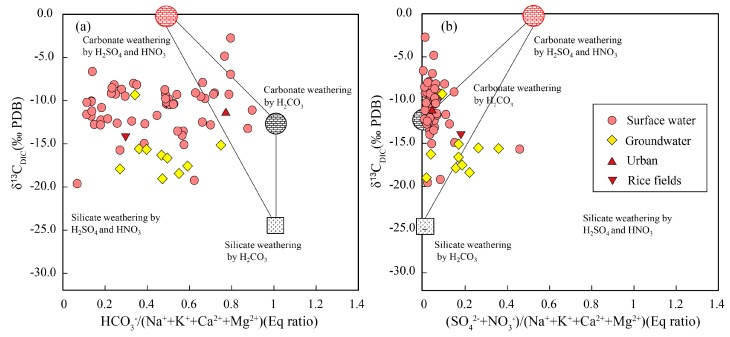
(**a**) δ^13^C_DIC_ vs. HCO_3_^−^/(Na^+^ + K^+^ + Ca^2+^ +Mg^2+^) and (**b**) (SO_4_^2−^ + NO_3_^−^)/(Na^+^ + K^+^ + Ca^2+^ Mg^2+^). Figure based on Liu, Xu, Sun, Zhao, Shi and Liu [48].

In this case, all carbon produced is derived from the carbonate and the δ^13^C_DIC_ value is similar to the carbonate minerals. Based on the above discussion, DIC from different weathering processes in the Mun River were plotted in Figure 5. Most water samples deviate from the three end-member mixing region and towards the silicate weathering by sulfuric and nitric acid area. Acid rain accelerates rock weathering and the role of the anthropogenic sourced acid (sulfuric and nitric acid) on silicate weathering is not negligible in the Mun River Basin.

### 4.3. Carbon Isotopic Composition of Anthropogenic DIC

**Figure 6:**
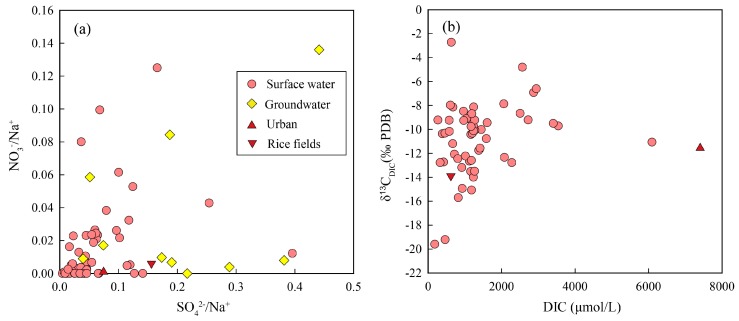
Plots of NO_3_^−^/Na^+^ vs. SO_4_^2−^/Na^+^ (**a**) and δ^13^C_DIC_ vs. DIC (**b**).

As one of the important agricultural production areas and population gathering place in Thailand, the chemistry of the Mun River water could be significantly impacted by human activities [8,48]. TDS reflects both the different lithologies drained by the river but also can be influenced by human activities on the water quality [6]. Higher TDS were mainly found in the middle reaches, which may be related to the dense population in the middle reaches. In addition, $K^{+}$, ${\mathrm{Na}}^{+}$, ${\mathrm{Cl}}^{-}$, ${\mathrm{NO}}_{3}^{-},$ and ${\mathrm{SO}}_{4}^{2-}$ are usually related to agricultural fertilizers, animal waste, and sewage in the river. If we focus on the mainstream, we can observe a sharp increase in ${\mathrm{Cl}}^{-}$, ${\mathrm{NO}}_{3}^{-},$ and ${\mathrm{SO}}_{4}^{2-}$ concentration in the middle of the river and this increase can mainly be related to the fertilizers used for agriculture. The case of chlorine is also characteristic but less obvious due to the contribution of evaporites dissolution in the study area. Calcium, ${\mathrm{Mg}}^{2+},$ and ${\mathrm{HCO}}_{3}^{-}$ are conventionally considered to be insensitive to human pollution [9]. To assess the impact of the urban and agricultural wastewater on river water, the waste water samples were collected from sewage pipe in Khorat Province and the rice field. Figure 6a shows the relationships between the molar ratios ${\mathrm{NO}}_{3}^{-}$/${\mathrm{Na}}^{+}$, ${\mathrm{SO}}_{4}^{2-}$/${\mathrm{Na}}^{+}$ for the river waters. Two wastewater samples were characterized by low ${\mathrm{NO}}_{3}^{-}$/${\mathrm{Na}}^{+}$ concentrations, and the values were approximately 0. The ${\mathrm{SO}}_{4}^{2-}$ may originate from various sources, such as oxidation of sulfides, the dissolution of gypsum, and acid deposition [41]. Higher DIC concentrations (7397 μmol/L) and medium δ^13^C_DIC_ value (−11.4‰) were found in the urban sewage sample (Figure 6b), and this large amount of untreated sewage discharged from rural areas may have a huge impact on concentrations of DIC and δ^13^C_DIC_. Shin, et al. [49] reported that δ^13^C_DIC_ values of detergents measured in three streams in a South Korean study ranged from −12.0 to −6.5‰. Therefore, in urban streams, detergent may be an important source of river water DIC.

### 4.4. DIC Evasion from the River System

Carbon dioxide will spread out of the river through the water-air interface when the *p*CO_2_ in the river water is greater than the partial pressure of carbon dioxide in the surrounding atmosphere. With the loss of CO_2_, the isotopic composition of the remaining DIC has changed accordingly [15]. In this study, carbon dioxide fluxes through the water-air interface were estimated using the following equation:

*F*_CO2_ = *k* × (C_water_ − C_air_) (4)

where *F*_CO2_ is the CO_2_ flux through the water-air interface, *k* is the gas transfer velocity (cm/h), C_water_ and C_air_ is the CO_2_ concentration in the water and air, respectively. C_water_ and C_air_ are typically calculated from the CO_2_ solubility, *K*_H_ (mol/m·atm), and the partial pressure of CO_2_ (*p*CO_2_, μatm) in the water (*p*CO_2w_) and air (*p*CO_2a_), respectively (i.e., C_w,a_ = *K*_H_ × *p*CO_2w,a_). Positive values of *F*_CO2_ represent fluxes from the water to air, and negative *F*_CO2_ values indicate CO_2_ invasion from air to water. The atmospheric CO_2_ concentration 445 ppmv was used here, and *K* is a temperature-dependent Schmidt number (ScT) for fresh water [13]:

*k* = *k*_600_ × (ScT/600) − 0.5 (5)

With

ScT = 1911.1 − 118.11T + 3.4527T^2^ − 0.04132T^3^ (6)

where T is the in-situ water temperature (°C), and *k*_600_ is the *K* for CO_2_ at 20 °C in freshwater. For small streams, we use *k*_600_ = 13.82 + 0.35w, where w is the water flow rate (cm/s). For the main channel (width >100 m), we use *k*_600_ = 4.46 + 7.11 × −u_10_. Where −u_10_ is the wind speed 10 m above rivers. Due to the lack of in-situ wind speed data, here the wind speed data of the nearest meteorological department were used to approximately replace the wind speed 10 m above the rivers.

**Figure 7:**
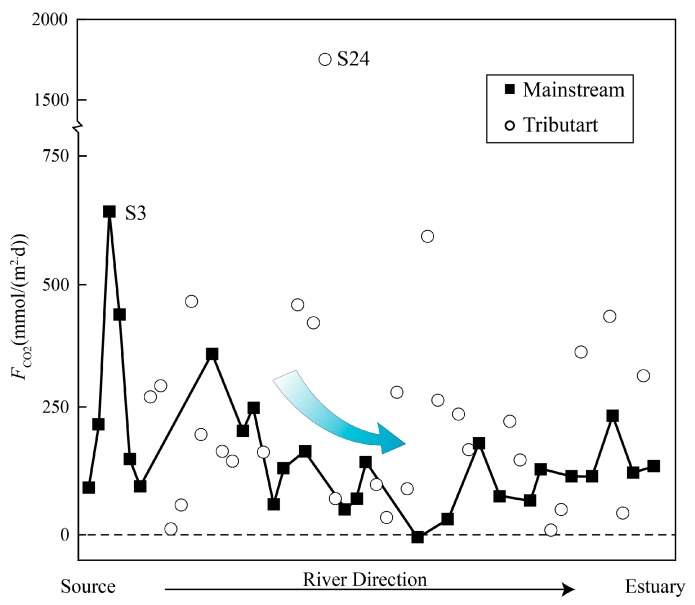
Spatial distributions of *F*_CO2_ in the Mun River.

Spatial changes occur in the direction and magnitude of *F*_CO2_ (Figure 7). The source region generally had high carbon dioxide flux. The evasion fluxes for the source region in this study show that the evasion of carbon from tropical rivers is not to be ignored. In the dry season, fluxes varied from −6 to 1826 mmol/(m^2^·d) with an average of 240 mmol/(m^2^·d). *F*_CO2_ values from the Mun River are similar to values from other tropic rivers (Table 1). The observed spatial differences, combined with the changes in other rivers around the world, indicate that carbon dioxide evasion in tropical rivers is huge but there are also large uncertainties.

## 5. Conclusions

Most of the surface and groundwater samples were dominated by ${\mathrm{Na}}^{+}$ and ${\mathrm{HCO}}_{3}^{-}$. The concentration of other cations (such as ${\mathrm{Mg}}^{2+}$ and ${\mathrm{Ca}}^{2+})$ is less than 50% of the sum of the major cations. For anions, the sum of ${\mathrm{SO}}_{4}^{2-}$, ${\mathrm{Cl}}^{-},$ and ${\mathrm{NO}}_{3}^{-}$ only contributes about 45% of the total anions in all the samples. In the surface water samples, the DIC content ranged from 185 to 5897 μmol/L (average 1376 μmol/L) in the Mun River. In most of the samples, bicarbonate was the main component of DIC, accounting for about 82% of DIC, and the rest is mainly composed of dissolved carbon dioxide due to it containing more acidic samples. Urban domestic sewage contains abnormally high DIC (7397 μmol/L), and ${\mathrm{HCO}}_{3}^{-}$ was the dominant species (92.4%).

The carbon isotopic compositions (δ^13^C_DIC_) of surface water ranged from −19.6‰ to −2.7‰. Urban sewage and farmland water did not show abnormal carbon isotope values (−11.4‰ and −13.8‰, respectively). In spite of the high variability in DIC concentrations and *p*CO_2_, the δ^13^C_DIC_ values of the groundwater were relatively consistent, with a mean value of −16.9 ± 1.4‰ (*n* = 9). Spatial changes occured in the direction and magnitude of *F*_CO2_. In the dry season, fluxes varied from −6 to 1826 mmol/(m^2^·d) with an average of 240 mmol/(m^2^·d). In addition to the dominant control on hydrochemistry and dissolved inorganic carbon isotope composition by the rock weathering, the impacts from anthropogenic activities were also observed in the Mun River, especially higher DIC concentration of waste water from urban activities. These human disturbances may affect accurate estimate contributions of carbon dioxide from tropical rivers to the atmospheric carbon budgets.

## Figures and Tables

**Table 1:** The average *p*CO_2_ and CO_2_ evasion flux of the Mun River and other rivers around the world.

River	Location	Climate	DIC	*p*CO_2_	*k*	*F*_CO2_	References
			mmol/L	μatm	cm/h	mmol/(m^2^·d)	
Mun	Thailand	Tropic	1.4	4392	10	240	This study
Lower Mekong	East Asia	Tropic	1.6	1090	26	195	[5]
Sinamay	French	Tropic	-	-	-	30–461	[50]
Amazon	Brazil	Tropic	-	4350	10	189.0	[15]
Amazon	Brazil	Tropic	-	3320	15	345.2	[51]
Nanpan	China	Subtropics	2.8	2644	8	194	[52]
Beipan	China	Subtropics	2.6	1287	8	78	[52]
Xijiang	China	Subtropics	1.6	2600	8–15	189–356	[53]
Yangtza	China	Subtropics	1.7	1297	-	14.2	[19]
Longchuan	China	Subtropics	1.1–4.6	1230–2100	-	74–156	[54]
Ottawa	Canada	Temperate	0.05–3	1200	4	80.8	[17]
Hudson	USA	Temperate	-	1125	4	16–s37	[18]
Mississippi	USA	Temperate	0.5	1335	-	270	[2]

## Supplementary Materials

The following are available online at https://www.mdpi.com/1660-4601/16/18/3410/s1, Table S1: The physical–chemical parameters and major ions concentration in the Mun River, Table S2: DIC system and carbon isotope composition of surface water and groundwater.
