# Supplementary material for: Hydrochemistry and Dissolved Inorganic Carbon (DIC) Cycling in a Tropical Agricultural River, Mun River Basin, Northeast Thailand
Source: Int J Environ Res Public Health. 2019 Sep 14;16(18):3410. doi: 10.3390/ijerph16183410 (PMC6765941; doi:10.3390/ijerph16183410)
Supplement: Supplementary file 1 [file ijerph-16-03410-s001.pdf]

SUPPLEMENTARY DATA

HYDROCHEMISTRY AND DISSOLVED INORGANIC CARBON (DIC) CYCLING IN A TROPICAL AGRICULTURAL RIVER, MUN RIVER BASIN, NORTHEAST THAILAND

Xiaoqiang Li, Guilin Han*, Man Liu, Kunhua Yang, Jinke Liu

Table S1 The physical-chemical parameters and major ions concentration in the Mun River.

Table S2 DIC system and carbon isotope composition of surface water and groundwater and CO_2 flux through water-air interface (F_{CO2}).

Table S1. The physical-chemical parameters and major ions concentration in the Mun River.

Samples	T (℃)	DO (mg/L)	ORP (mV)	EC (μs/cm)	pH	Na^+ (mg/L)	K^+	Ca^{2+}	Mg^{2+}	Cl^-	SO_4^{2-}	NO_3^-	SiO_2	Alk μmol/L	TDS[a] (mg/L)	TZ^+ (ueq/L)	TZ^-	NICB[b] (%)
S1	29.8	10.3	251.6	88.0	7.4	3.5	2.8	5.9	2.7	5.7	0.6	0.0	4.3	585	56	749	756	-0.9
S2	28.3	7.2	265.1	72.5	7.0	2.6	3.1	4.6	2.1	4.5	1.4	0.0	5.3	465	47	599	620	-3.5
S3	28.0	3.3	207.1	171.1	6.8	14.6	4.4	8.2	4.4	18.7	2.7	0.9	9.6	870	112	1532	1469	4.1
S4	26.4	5.9	288.7	293.9	7.3	16.0	5.5	24.8	10.3	14.5	17.0	1.8	11.9	2040	194	2929	2831	3.4
S5	28.2	5.1	180.7	348.4	7.5	39.5	10.6	10.9	5.3	49.9	13.0	4.1	10.5	1320	224	3006	3064	-1.9
S6	26.5	6.2	250.4	252.3	7.5	26.8	8.2	10.4	4.5	35.3	13.9	3.8	12.2	870	167	2271	2217	2.4
S7	28.3	6.8	235.4	101.9	7.1	4.6	2.3	9.3	2.0	2.8	2.3	0.4	14.4	780	66	899	912	-1.4
S8	26.7	7.2	268.1	631.0	7.9	14.1	2.6	103.5	9.6	19.2	9.8	4.8	19.0	5940	412	6641	6762	-1.8
S9	28.8	11.8	228.4	344.3	8.5	18.1	4.9	38.0	7.1	19.2	9.0	0.3	3.6	2595	220	3403	3329	2.2
S10	26.0	7.4	206.4	347.9	8.2	17.7	5.0	42.4	7.1	18.2	8.5	0.2	4.5	2850	232	3602	3544	1.6
S11	26.2	7.0	287.0	507.0	7.4	38.8	7.7	46.9	8.1	45.6	15.6	2.7	7.8	3285	335	4900	4941	-0.8
S12	26.7	7.2	273.2	515.0	7.8	41.1	7.1	48.3	8.3	48.8	17.5	2.4	8.3	3300	412	5066	5077	-0.2
S13	27.6	5.6	279.7	640.0	7.3	84.0	12.7	22.5	8.7	126.7	16.1	0.6	12.2	1890	411	5823	5809	0.2
S14	29.8	8.2	261.5	2452.0	7.8	369.6	6.9	62.5	16.2	668.5	6.5	0.0	13.5	2865	1502	20704	21859	-5.6
S15	26.8	6.6	270.9	300.0	7.7	26.3	5.1	22.1	7.5	27.6	4.6	0.0	11.3	1980	197	3000	2855	4.9
S16	28.4	7.4	279.0	980.0	7.7	134.8	14.1	30.0	11.6	215.7	18.3	0.0	8.0	2400	618	8677	8866	-2.2
S17	30.5	6.9	260.1	1270.0	7.7	169.3	9.7	38.7	11.4	256.0	7.9	0.0	14.2	2610	768	10481	9997	4.6
S18	28.1	5.2	287.3	312.7	7.4	26.6	5.2	18.6	5.5	56.4	2.2	0.4	11.9	1035	202	2684	2676	0.3
S19	28.2	7.6	262.2	626.0	7.9	81.5	5.1	22.7	6.7	126.2	22.6	0.0	11.7	1575	397	5364	5607	-4.5
S20	28.3	6.5	261.7	951.0	7.6	141.2	6.1	27.6	7.9	235.1	17.5	1.1	11.9	1515	598	8327	8531	-2.4
S21	26.5	3.8	206.0	165.0	6.9	19.3	2.5	8.7	3.3	18.2	1.9	1.2	11.2	915	111	1614	1487	7.9
S22	28.1	5.2	51.4	1115.0	7.5	169.3	5.7	32.4	7.4	282.1	31.3	3.2	17.3	1290	700	9783	9949	-1.7
S23	31.3	5.7	323.0	291.7	6.9	31.8	4.8	14.8	1.9	16.8	52.5	1.0	21.3	645	178	2413	2229	7.6
S24	29.9	3.9	333.7	90.2	6.1	9.1	2.0	5.6	1.2	8.9	1.2	0.3	6.7	465	57	833	748	10.2
S25	26.2	6.3	262.4	277.8	7.2	21.5	2.6	25.6	3.2	68.0	3.2	0.2	4.9	375	185	2552	2364	7.4
S26	24.0	8.2	266.5	1128.0	7.9	187.9	5.1	30.2	8.4	330.7	17.2	0.3	10.7	1410	770	10500	11102	-5.7
S27	25.6	6.5	238.6	1308.0	7.8	213.7	5.1	43.3	8.4	361.6	40.8	1.2	13.3	1365	863	12285	12434	-1.2
S28	26.9	6.0	285.0	995.0	7.5	161.5	4.0	23.8	6.2	270.4	13.7	0.7	12.4	1185	644	8825	9110	-3.2
S29	25.5	6.4	310.0	215.6	6.6	36.1	1.7	1.3	1.5	58.0	2.2	0.2	4.6	120	146	1808	1806	0.1
S30	26.7	7.1	261.2	544.0	7.7	86.7	2.9	11.2	3.9	146.2	3.3	0.0	6.9	645	356	4730	4838	-2.3

Samples	T (℃)	DO (mg/L)	ORP (mV)	EC (μs/cm)	pH	Na^{+} (mg/L)	K^{+}	Ca^{2+}	Mg^{2+}	Cl^{-}	SO_4^{2-}	NO_3^{-}	SiO_2	Alk μmol/L	TDS[a] (mg/L)	TZ^{+} (ueq/L)	TZ^{-}	NICB[b] (%)
S31	31.2	7.7	335.8	77.9	6.7	6.7	1.5	3.1	0.8	10.9	1.1	0.0	7.8	270	49	558	601	-7.7
S32	26.8	4.7	217.7	610.0	7.6	92.6	3.3	17.0	5.0	159.0	2.9	0.2	9.5	960	398	5379	5508	-2.4
S33	26.7	8.7	245.3	566.0	8.5	82.5	3.4	18.0	5.1	136.5	5.7	0.0	7.1	1140	372	5002	5109	-2.1
S34	31.2	7.0	338.6	65.6	6.3	4.3	2.2	2.2	1.2	8.8	0.4	0.0	7.3	225	41	463	481	-4.1
S35	30.1	7.6	288.9	290.9	7.0	37.1	3.5	8.3	2.2	54.4	5.6	8.0	7.3	555	181	2302	2337	-1.5
S36	29.3	7.7	249.5	480.0	7.9	67.5	3.0	14.4	4.3	107.3	2.5	0.0	8.8	945	300	4088	4024	1.6
S37	28.4	4.1	251.4	186.6	7.2	8.4	6.8	9.9	3.2	18.3	3.5	1.4	12.6	930	121	1483	1541	-3.9
S38	29.6	7.5	284.2	189.3	7.1	20.6	3.0	7.7	2.6	34.5	1.9	0.3	10.7	525	119	1582	1543	2.5
S39	30.4	6.3	268.0	505.0	7.4	64.4	3.8	19.2	5.1	108.7	4.4	2.8	8.5	1095	310	4286	4298	-0.3
S40	28.8	6.8	289.5	242.0	7.2	22.2	3.4	17.4	3.6	27.3	5.8	1.5	8.3	1155	155	2219	2070	6.7
S41	28.9	6.4	245.2	246.8	7.7	22.7	3.4	17.3	3.6	28.2	5.7	1.6	8.4	1125	157	2234	2063	7.6
S42	30.6	7.1	294.7	354.0	7.1	50.7	1.5	8.5	3.0	73.4	5.4	0.0	12.7	615	218	2918	2799	4.1
S43	31.0	7.2	303.0	564.0	7.2	88.6	1.7	8.9	2.5	136.8	12.7	0.0	13.3	510	343	4553	4634	-1.8
S44	29.3	6.3	233.8	253.0	7.8	22.9	3.4	17.1	3.6	29.7	5.6	1.4	8.2	1170	160	2236	2147	4.0
S45	29.3	6.2	252.1	250.1	7.6	21.9	3.5	17.5	3.7	27.5	6.0	0.0	8.5	1200	158	2219	2099	5.4
S46	28.7	6.5	203.3	211.0	7.7	31.6	1.3	3.8	2.2	48.7	1.9	0.2	7.5	315	136	1782	1732	2.8
S47	29.8	7.8	222.1	393.2	7.9	45.4	3.3	21.1	4.5	63.3	10.3	0.8	9.9	1215	245	3487	3229	7.4
S48	29.9	6.1	249.6	264.0	7.6	24.2	3.6	18.3	3.8	30.1	6.5	1.5	8.3	1185	166	2380	2192	7.9
S49	29.4	4.0	287.0	115.2	6.9	6.3	6.3	7.9	1.8	9.9	1.2	0.0	7.9	645	75	982	949	3.4
S50	30.4	7.1	248.7	278.1	7.6	26.2	3.7	19.1	3.9	32.5	6.8	1.5	8.4	1215	173	2516	2298	8.7
S51	29.7	6.2	330.3	47.1	6.5	3.7	1.6	3.5	0.7	2.7	1.1	1.0	5.7	270	31	444	385	13.3
S52	30.4	6.7	263.1	258.3	7.3	24.5	3.5	17.3	3.6	30.4	6.3	1.6	7.7	1131	160	2320	2147	7.5
S53	33.0	7.9	221.3	216.9	7.9	16.3	2.3	15.6	2.7	23.5	3.0	0.5	7.9	954	130	1778	1687	5.1
S54	30.6	8.0	227.1	263.8	7.6	24.8	3.5	17.5	3.7	31.5	5.9	1.3	7.6	1131	163	2352	2164	8.0
S55	30.1	7.8	298.0	22.9	6.3	1.3	1.2	1.1	0.5	1.7	0.8	0.0	4.4	129	15	185	194	-5.2
S56	29.2	7.2	225.4	253.7	7.5	24.1	3.3	17.0	3.6	30.9	5.5	1.5	7.5	1104	161	2283	2115	7.4
S57	27.7	7.6	172.6	263.8	7.6	9.0	2.5	26.9	8.8	16.0	17.8	1.0	10.0	1584	50	2532	2421	4.4
W2	27.0	1.6	-109.4	937.0	7.4	71.7	8.1	95.2	11.6	73.7	20.8	0.0	24.9	6850	605	9267	9363	-1.0
W1	29.1	7.4	228.6	198.4	7.0	22.0	3.7	8.3	2.5	27.9	14.7	0.2	14.2	510	126	1678	1605	4.4
G1	24.9	5.9	256.9	361.5	6.8	51.0	5.3	20.9	3.7	84.1	10.9	8.0	7.0	1260	229	3699	3988	-7.2
G2	24.6	4.5	351.8	47.4	4.7	3.0	0.3	2.9	1.1	8.3	2.3	0.7	14.6	100	36	372	394	-5.7

Samples	T (℃)	DO (mg/L)	ORP (mV)	EC (μs/cm)	pH	Na^+ (mg/L)	K^+	Ca^{2+}	Mg^{2+}	Cl^-	SO_4^{2-}	NO_3^-	SiO_2	Alk μmol/L	TDS[a] (mg/L)	TZ^+ (ueq/L)	TZ^-	NICB[b] (%)
G3	24.3	6.3	268.0	1587.0	6.7	54.1	2.0	163.2	90.9	333.6	16.7	2.5	5.1	8475	927	18026	18274	-1.4
G4	24.8	3.7	107.5	217.4	5.8	20.7	0.9	11.7	4.5	38.5	3.4	0.5	16.2	875	123	1878	2039	-7.9
G5	24.1	4.1	38.0	2075.0	6.8	226.6	1.6	339.1	19.5	355.1	360.9	4.9	28.9	10225	1648	28417	27841	2.1
G6	24.0	3.4	51.5	1195.0	6.8	160.0	1.4	85.9	36.2	78.3	115.7	4.2	32.7	10650	839	14256	15336	-7.0
G7	24.1	5.3	143.2	183.9	6.2	20.2	2.5	16.2	3.0	16.2	18.3	0.0	66.7	1175	179	1995	2014	-0.9
G8	24.4	6.0	96.9	1256.0	7.1	142.5	65.7	97.2	13.5	196.5	113.3	2.6	63.3	6825	903	13834	14769	-6.3
G9	24.7	3.7	113.0	841.0	7.0	52.0	0.4	78.4	48.9	54.9	95.7	19.1	120.1	5600	640	10204	9449	8.0
G10	24.8	3.7	116.4	3166.0	7.3	573.0	42.6	162.8	69.8	284.9	690.3	6.1	70.1	15750	2380	39869	38266	4.2

[a] TDS: total dissolved solid

[b] NICB: Normalized Inorganic Charge Balance, NICB = $(TZ^+ - TZ^-) / TZ^- \times 100$

Table S2 DIC system and carbon isotope composition of surface water and groundwater and CO_2 flux through water-air interface (F_{CO2}).

Samples	DIC μmol/L	HCO_3 μmol/L	CO_2 μmol/L	CSI	pCO_2 μatm	F_{CO2} mmol/(m^2·d)	$\delta^{13}C$ (‰)	SE (‰)
S1	633	583	49	-1.6	1930	94	-2.7	0.02
S2	575	465	110	-2.2	4158	221	-9.2	0.02
S3	1183	870	313	-1.9	11520	646	-15.1	0.01
S4	2280	2036	241	-0.7	8297	440	-12.8	0.03
S5	1399	1315	82	-0.9	2979	151	-11.7	0.01
S6	929	867	60	-1.1	2117	96	-14.9	0.02
S7	916	779	137	-1.6	5123	277	-13.2	0.01
S8	6093	5897	175	0.9	5691	297	-11.1	0.04
S9	2566	2509	17	0.8	580	11	-4.8	0.02
S10	2874	2811	44	0.6	1460	59	-6.9	0.03
S11	3544	3276	263	-0.1	8804	466	-9.7	0.01
S12	3407	3280	117	0.3	3937	199	-9.5	0.02
S13	2080	1886	192	-0.7	6696	362	-12.3	0.01
S14	2946	2845	92	0.3	3112	166	-6.6	0.01
S15	2061	1970	86	-0.3	2999	147	-7.9	0.02
S16	2507	2389	113	-0.2	3926	208	-8.7	0.02
S17	2726	2597	123	0.0	4464	255	-9.2	0.01
S18	1122	1032	89	-0.9	3217	165	-12.7	0.01
S19	1608	1561	41	-0.2	1432	61	-9.4	0.01
S20	1588	1508	76	-0.4	2665	133	-10.8	0.01
S21	1163	914	248	-1.8	8777	468	-13.5	0.02
S22	1382	1286	94	-0.6	3254	167	-11.8	0.01
S23	823	644	178	-1.7	7038	427	-15.7	0.03
S24	1234	466	769	-3.0	30150	1826	-14.0	0.04
S25	423	374	49	-1.4	1683	72	-12.7	0.02
S26	1449	1400	44	-0.3	1349	50	-10.0	0.02
S27	1413	1357	53	-0.2	1668	70	-11.6	0.02
S28	1271	1181	88	-0.8	2961	145	-10.1	0.03
S29	185	120	64	-3.8	2224	100	-19.6	0.01
S30	672	641	29	-1.1	993	34	-11.2	0.04
S31	389	270	118	-2.9	4831	284	-10.4	0.02
S32	1017	956	59	-0.9	2033	92	-12.2	0.04
S33	1130	1106	8	0.1	283	-6	-9.1	0.01
S34	463	225	237	-3.5	9705	596	-10.3	0.04
S35	678	554	123	-1.9	4741	269	-8.1	0.03
S36	965	936	25	-0.6	908	31	-8.5	0.02
S37	1050	928	121	-1.4	4491	241	-9.1	0.02
S38	607	524	83	-1.8	3182	170	-8.0	0.02
S39	1181	1092	88	-0.8	3329	183	-12.6	0.02
S40	1296	1153	143	-1.0	5296	291	-10.4	0.02
S41	1167	1118	46	-0.6	1699	78	-10.4	0.01
S42	718	614	103	-1.7	3998	226	-12.1	0.03
S43	580	509	71	-1.7	2735	149	-10.2	0.01
S44	1206	1162	40	-0.5	1509	67	-10.3	0.01

S45	1266	1195	69	-0.7	2581	132	-13.5	0.02
Samples	DIC μmol/L	HCO_3 μmol/L	CO_2 μmol/L	CSI	pCO_2 μatm	F_{CO2} mmol/(m²·d)	$\delta^{13}C$ (‰)	SE (‰)
S46	328	313	14	-1.8	540	9	-12.8	0.03
S47	1241	1204	32	-0.3	1205	50	-8.1	0.02
S48	1242	1179	60	-0.6	2285	116	-9.8	0.01
S49	810	644	166	-1.9	6380	362	-12.5	0.06
S50	1270	1209	59	-0.6	2250	116	-9.2	0.01
S51	465	270	195	-3.0	7689	445	-19.2	0.01
S52	1239	1128	109	-0.9	4210	238	-10.1	0.03
S53	973	944	25	-0.4	1013	43	-9.3	0.04
S54	1189	1126	61	-0.7	2366	124	-8.7	0.03
S55	267	129	138	-4.0	5531	317	-9.2	0.14
S56	1173	1100	71	-0.8	2663	137	-9.8	0.02
S57	1669	1577	88	-0.4	3138	158	-9.1	0.01
W2	7397	6832	557	0.5	18320	1014	-11.4	0.03
W1	632	510	123	-2.0	4636	254	-13.8	0.03
G1	17551	1259	409	-1.4	13520	-	-9.3	0.02
G2	17551	120	5404	-5.3	167700	-	-17.9	0.01
G3	17551	8472	4201	-0.1	121300	-	-19.0	0.02
G4	17551	877	3129	-2.8	105100	-	-16.3	0.03
G5	17551	10219	3597	0.4	100900	-	-15.5	0.01
G6	17551	10645	4307	-0.1	128500	-	-15.1	0.05
G7	17551	1175	1647	-2.1	54120	-	-17.5	0.02
G8	17551	6817	1171	0.1	35450	-	-16.6	0.00
G9	17551	5595	1321	-0.2	40650	-	-18.4	0.01
G10	17551	15721	1816	0.7	51590	-	-15.6	0.03
